# High-throughput sequencing discovered diverse monopartite and bipartite begomoviruses infecting cucumbers in Saudi Arabia

**DOI:** 10.3389/fpls.2024.1375405

**Published:** 2024-10-10

**Authors:** Muhammad Naeem Sattar, Mostafa I. Almaghasla, Muhammad Nouman Tahir, Sherif M. El-Ganainy, Biju Vadakkemukadiyil Chellappan, Muhammad Arshad, Nizar Drou

**Affiliations:** ^1^ Central Laboratories, King Faisal University, Al-Ahsa, Saudi Arabia; ^2^ Department of Arid Land Agriculture, College of Agricultural and Food Sciences, King Faisal University, Al-Ahsa, Saudi Arabia; ^3^ Pests and Plant Diseases Unit, College of Agriculture and Food Sciences, King Faisal University, Al-Ahsa, Saudi Arabia; ^4^ Department of Plant Protection, Faculty of Agricultural Sciences, Ghazi University, Dera Ghazi Khan, Pakistan; ^5^ Plant Pathology Research Institute, Agricultural Research Center, Giza, Egypt; ^6^ Department of Biological Sciences, College of Science, King Faisal University, Al-Ahsa, Saudi Arabia; ^7^ Bioinformatics Core, Center for Genomics and Systems Biology, New York University Abu Dhabi, Abu Dhabi, United Arab Emirates

**Keywords:** cucumber plants, geminiviruses, genetic diversity, Illumina MiSeq, mixed infection, Saudi Arabia

## Abstract

Limited research in Saudi Arabia has devolved into the prevalence and genetic diversity of begomoviruses. Utilizing Illumina MiSeq sequencing, we obtained 21 full-length begomovirus sequences (2.7–2.8 kb) from eight cucumber plants grown in fields and greenhouses. We found that two complete begomovirus genomes were variants of the Boushehr strain of tomato yellow leaf curl virus (TYLCV) with nucleotide (nt) sequence identities of 94.7-95.9%. Another full-length genome was a variant of TYLCV-Iran with 94.6% identity. Five full-length sequences closely matched the DNA-A of watermelon chlorotic stunt virus (WmCSV) isolates with 97.9-98.7% nt sequence identities, while five sequences had their highest nt sequence identities (95.8-96.3%) with the DNA-B of WmCSV isolates. Simultaneously, four sequences were 99.1-99.6% identical to the DNA-A of tomato leaf curl Palampur virus (ToLCPalV). Four sequences matched the DNA-B of ToLCPalV reported from Iran and Saudi Arabia with identities ranging from 96.2-100%. Four plants showed a mixed infection of these begomoviruses. Most ORFs showed evidence of negative selection pressure, suggesting that purifying selection plays a crucial role in shaping the diversity of these begomoviruses. Additionally, potential intra- and interspecies recombination events were detected in the TYLCV and WmCSV DNA-B genomic regions. The ToLCPalV isolates identified in this study formed a cluster with the other ToLCPalV isolates reported from Saudi Arabia, Iran and Iraq, representing a unique lineage distinct from ToLCPalV reported from Southeast Asia. High mutation rate and robust selection facilitated the independent evolution of ToLCPalV without recombination. Overall, this study offers valuable insights into the diversity and evolutionary dynamics of begomoviruses infecting cucumber crops in Al-Ahsa, Saudi Arabia.

## Introduction

Single-stranded DNA (ssDNA) viruses of the family *Geminiviridae*, encompassing either one or two viral components encapsulated within geminate icosahedra, serve as causal agents of plant diseases prevalent in tropical and subtropical regions (Zerbini et al., 2017). The *Geminiviridae* family, comprising 14 genera, holds the distinction of being the largest family of plant viruses. These viruses are capable of causing devastating yield losses in both monocotyledonous and dicotyledonous crops (Fiallo-Olivé et al., 2021). With over 500 species, the *Geminiviridae* family boasts an extensive repertoire of viruses. The *Begomovirus* genus, with its two lineages, Old World (OW) and New World (NW), stands out as the most species-rich group (Fiallo-Olivé and Navas-Castillo, 2020). Begomoviruses exhibit two genome configurations: bipartite, consisting of two components (DNA-A and DNA-B) each approximately 2.6 kb, and monopartite, featuring a single component (DNA-A) similar to that of the bipartite counterpart ranging from 2.7-2.8 kb. While the majority of begomoviruses conform to a specific genomic structure based on their geographic origin, there are intriguing exceptions. Notably, there are only a limited number of instances of bipartite begomoviruses indigenous to the OW, and conversely, monopartite begomoviruses are rarely found native to the NW (Romay et al., 2019).

In the OW, both monopartite begomoviruses and the DNA-A component of bipartite begomoviruses harbor six open reading frames (ORFs). Two genes encoded by the virion-sense DNA strand include the coat protein (CP) and the AV2/V2 protein, while four genes encoded by the complementary-sense DNA strand include the replication-associated protein (Rep), the transcriptional activator protein (TrAP), the replication enhancer protein (REn), and the C4 protein (Hanley-Bowdoin et al., 2013). Besides the six established ORFs, recent studies have identified a set of novel small ORFs encoded within the monopartite begomovirus genome, including V3, C5, C6, and C7 (Gong et al., 2021; Liu et al., 2023). The DNA-B component of bipartite begomoviruses also possesses two ORFs, the nuclear shuttle protein (NSP; BVI) encoded by the virion-sense DNA strand and the movement protein (MP; BC1) encoded by the complementary-sense DNA strand (Fondong, 2013). Bipartite begomoviruses possess a common region (CR) spanning around 200 nucleotides (nt) in both DNA-A and DNA-B. This CR, housing the origin of replication, is a shared non-coding segment with a nona-nucleotide sequence (TAATATTAC). This CR is indispensable for replication and bidirectional gene expression. In the OW, many of the economically important plant diseases are caused by monopartite begomoviruses in association with single-stranded DNA-satellite components (betasatellites, alphasatellites, and deltasatellites) (Fiallo-Olivé and Navas-Castillo, 2020). Betasatellites, in particular, influence disease development by encoding the symptom determinant protein βC1 (Yang et al., 2019). Recently, another ORF βV1 has also been shown to act as a protein elicitor and pathogenicity determinant (Gupta et al., 2022). Alphasatellites and deltasatellites play crucial roles in the context of monopartite begomovirus infection by providing additional functions that contribute to the complexity of the infection process (Briddon et al., 2018).

The widespread distribution of begomoviruses is attributed to the widespread prevalence of the cryptic species complex of whiteflies (*Bemisia tabaci*), non-cultivated host plants, human activity, and the ability of the virus to evolve through recombination and the acquisition of new components (Gilbertson et al., 2015; Rojas et al., 2018). While the indigenous begomovirus species within a region exhibit genetic divergence from introduced species, they can evolve in parallel or through localized adaptations (Zhou et al., 2008). Intercontinental viral migrations have become increasingly evident in recent years. Mabvakure et al. (2016) observed the westward spread of tomato yellow leaf curl virus (TYLCV) across Africa in the 1990s, while Sobh et al. (2012) documented the arrival of northwestern squash leaf curl virus in the Middle East. Notable, Sattar et al. (2013) tracked the eastward expansion of cotton leaf curl disease (CLCuD) from the Indo-Pakistani subcontinent to China. More recently, OW watermelon chlorotic stunt virus (WmCSV) has made a dramatic cross-continental leap, reaching Mexico and the USA (Dominguez-Duran et al., 2018; Fontenele et al., 2021). Another noteworthy case is the identification of tomato leaf curl Palampur virus (ToLCPalV) in cucurbits, tomatoes, and melons in Iran (Heydarnejad et al., 2009) and most recently in Saudi Arabia (AlHudaib et al., 2022), indicating long-distance spread of begomoviruses. The ToLCPMV epidemic has inflicted severe damage on cucurbit production in Iran, leading to substantial economic losses for protected farms cultivating cucumbers (Heydarnejad et al., 2013).

Various monopartite and bipartite begomoviruses have been documented across the Arabian Peninsula, causing infections in diverse crops. Several begomoviruses pose significant threats to various vegetable crops in Saudi Arabia, with prominent examples including TYLCV, tomato leaf curl Sudan virus (ToLCSDV), WmCSV (Sattar, 2024), and cotton leaf curl Gezira virus (CLCuGeV) (Idris et al., 2014; Rezk et al., 2019; Sohrab, 2020). Most recently, ToLCPalV was first reported during a field survey conducted in the Al Ahsa region, in a mixed infection involving tomatoes and cucurbits (AlHudaib et al., 2022).

Cucurbits, belonging to the family *Cucurbitaceae*, are highly esteemed as vegetable crops. Among them, cucumber (*Cucumis sativus* L.) emerges as a noteworthy vegetable cultivated worldwide, boasting an estimated annual global production of approximately 93 million tons. In Saudi Arabia, a diverse range of vegetable crops is cultivated, with cucumber being extensively grown (Elzaki et al., 2022). In 2021, it covered an area of 2,357 hectares and yielded 0.18 million tons (https://www.indexbox.io/search/production-cucumber-and-gherkin-saudi-arabia/). Cucumbers are among the top choices for greenhouse cultivation in Saudi Arabia, occupying the second-highest position among all greenhouse vegetables (Mousa et al., 2019). During a survey of cucumber plants in both fields and greenhouses across the Al Ahsa region of Saudi Arabia, symptomatic plants were identified. Preliminary testing confirmed that these plants were infected with begomovirus. Despite considerable research efforts in this domain, conventional contemporary methods for detecting and characterizing the plant virome offer limited insights into the prevalent viral genomes (Idris et al., 2014; Shahid et al., 2021). Conventional approaches face obstacles in accurately evaluating the genetic diversity of begomoviruses within infected hosts due to the inherent selectivity of primers and the prevalence of particular viral genomes. The widespread importance of cucumber cultivation in Saudi Arabia prompted a comprehensive investigation into the potential of begomovirus infection on a broader scale, utilizing a next-generation sequencing approach. The study identified bipartite begomovirus WmCSV isolates from the symptomatic samples in mixed infections with both monopartite begomovirus TYLCV and bipartite begomovirus ToLCPalV. These mixed infections could involve either a single begomovirus species or both together.

## Materials and methods

### Plant samples collection and initial screening for begomovirus detection

**Figure 1:**
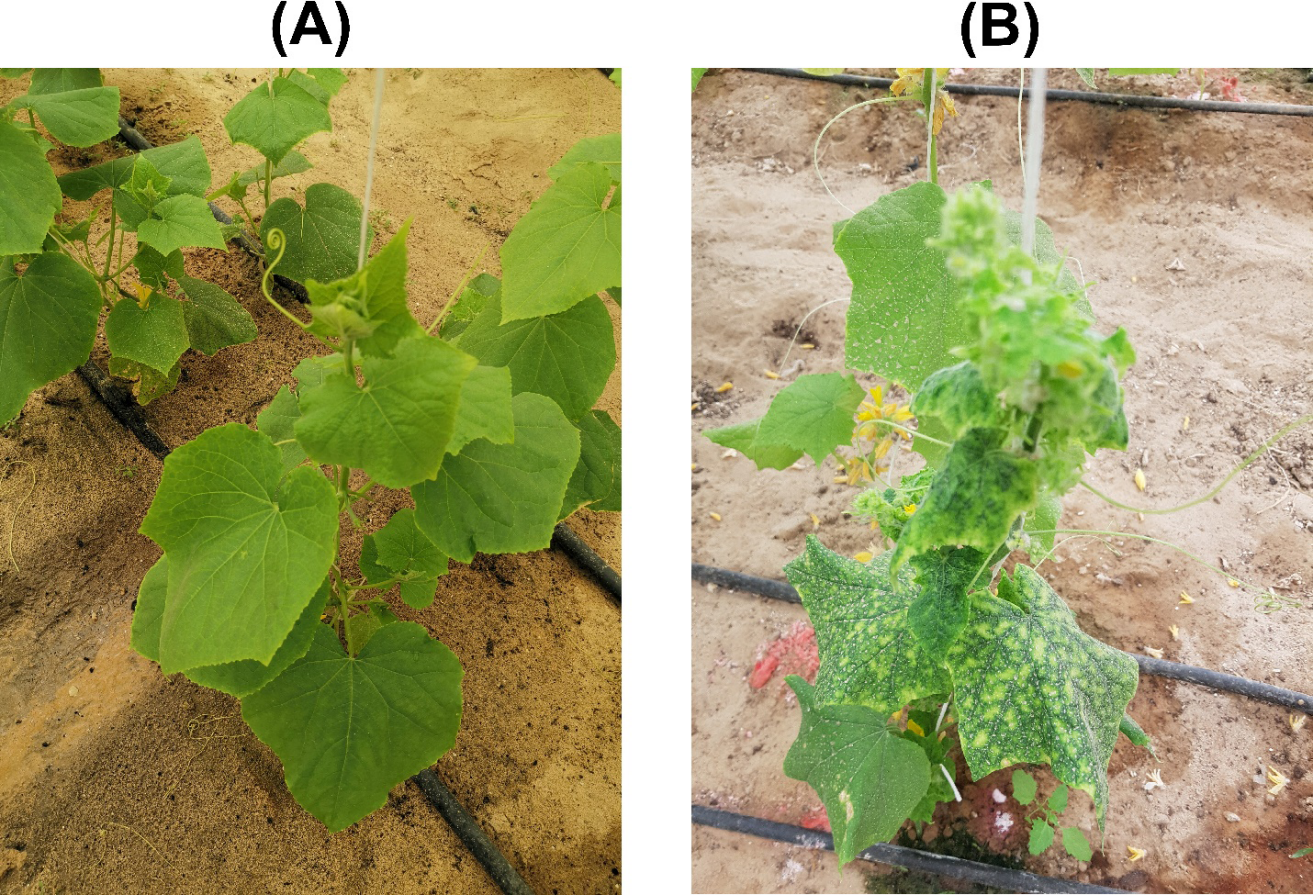
Symptoms of virus infection observed on plants in the Al-Ahsa region, Saudi Arabia: **(A)** a healthy cucumber plant, and **(B)** symptomatic cucumber plant exhibiting leaf yellowing and mosaic patterns.

Leaf samples displaying symptoms such as leaf yellowing and yellow mosaic patterns (**Figure 1**), indicative of begomovirus infection, were collected from seventeen cucumber (*Cucumis sativus* L.) plants in Al-Hofuf and Qateef regions, Saudi Arabia, across five different field plots and/or greenhouses. After collection, all samples were plunged into liquid nitrogen and stored at -80°C for future use. Genomic DNA extraction was performed from the samples using the DNeasy^®^ Plant Mini Kit (Qiagen, Germantown, MD, USA). An initial standardized polymerase chain reaction (PCR) was carried out using universal degenerate primers AC1048/AV494, targeting a ~550 bp fragment of the begomovirus core coat protein (CP) region (Wyatt and Brown, 1996) from all collected samples. Following the purification of amplicons using the GeneJet PCR purification kit from ThermoFisher Scientific (Waltham, MA, USA), PCR amplified products were sent for Sanger’s sequencing at Macrogen Korea sequencing facility.

### Deciphering full-length begomovirus genomes through rolling circle amplification and next-generation sequencing

To decipher complete sequences of begomovirus DNA molecules, rolling circle amplification (RCA) technique using Φ-29 DNA polymerase was performed on the genomic DNA from the leaf samples of eight cucumber plants using a commercial amplification kit (GE Healthcare, Chicago, IL, USA) (**Supplementary Table 1**). The purified RCA products underwent whole-genome *de novo* sequencing for begomovirus. However, it’s important to note that this NGS workflow is not suitable for identifying RNA viruses in the samples. The sequencing data were generated using the Nextera XT library preparation method and the Illumina MiSeq 300 bp PE platform at Macrogen, Korea.

### Begomovirus genome assembly and NGS data analysis

The quality of raw FASTQ-sequenced reads was initially assessed using FastQC (v0.11.8) (Andrews, 2022). Subsequently, the Trimmomatic tool v0.39 was employed for quality trimming (Bolger et al., 2014) and adapter sequence removal, utilizing the parameters as described earlier (AlHudaib et al., 2022). This step involved removing low-quality bases and adapter sequences that could introduce errors in downstream analyses. After the quality-trimming step, the reads were reassessed using FastQC to ensure acceptable quality for subsequent analyses. The quality-filtered reads were aligned against the corresponding reference genomes of the following begomoviruses: TYLCV (GU076454), ToLCPalV DNA-A (EU547683) and DNA-B (EU547681), WmCSV DNA-A (KJ939448) and DNA-B (KJ939447). Alignment of reads from all samples was performed using BWA-MEM2 v2.2.1 (Vasimuddin et al., 2019). Default parameters were employed, along with additional options (−k 10 and T 12). Following alignment, the SAM files were converted to the compact BAM format and subsequently sorted using SAMtools version 1.9 [68]. To ensure consistency and standardization of the aligned data, the Picard Tools pipeline (http://broadinstitute.github.io/picard/) was employed to assign all reads to a new read group within the output BAM file. Subsequently, consensus sequences for each begomovirus genomic component were assembled using mpileup function of SAMtools and piping the output to iVar consensus v1.3 (Umair et al., 2021). The Samtools pileup command was employed to generate a variant pipleup from the BAM files, incorporating specific parameters such as orphan read pairs (−A) and setting the minimum base quality for mapping to 0 (−Q 0). The iVar consensus command was executed using default parameters, with the exception of a minimum depth of 20 (−m 20) required consensus calling.

### PCR-mediated confirmation of the identified begomovirus genomic components

To confirm the presence of each begomovirus genomic component, the RCA products derived from the cucumber plant samples underwent a 1:10 dilution and served as templates for PCR reactions employing specific primers listed in **Supplementary Table 2**. The amplified PCR products were subsequently purified and subjected to Sanger sequencing at Macrogen for partial characterization. The identity of the obtained sequences was validated by non-redundant comparison with begomovirus sequences in the NCBI GenBank database.

### Nucleotide sequence alignments and measuring pairwise identities

The assessment of nt sequence identities for genomic components was commenced by utilizing the BLASTn tool within the NCBI GenBank database. Subsequently, the most relevant BLASTn results from the database were retrieved and used to quantify pairwise nt sequence identities for each component individually. Subsequently, to facilitate comparison, full-length nt sequences of each component were aligned using the ClustalW algorithm, implemented within the MEGA-11 software suite (Tamura et al., 2021). Following the established guidelines for geminivirus demarcation, the recommended Species Demarcation Tool (SDTv1.2) was employed to estimate the pairwise nt sequence identities (Muhire et al., 2013). The NCBI ORF finder tool was utilized to conduct a comparative analysis of individual ORFs and non-translated regions (NTRs) within the genomic components (https://www.ncbi.nlm.nih.gov/orffinder/, accessed on November 15, 2022).

### Phylogenetic dendrograms to decipher evolutionary relationships of begomovirus components

The evolutionary relationships of individual genomic components were elucidated by constructing phylogenetic dendrograms using the MEGA11 software. For each dataset, we employed the maximum likelihood method to estimate evolutionary distances, and a best-fitted gamma distribution with invariant sites (G+I) model to capture site-specific variation rates. The resulting phylogenetic trees were exported in EMF format and further enhanced for clarity using image-editing tool Adobe Illustrator (CC) 2021.

### Determining recombination hotspots in begomovirus genomic sequences

A comprehensive dataset comprising 400 complete begomovirus DNA-A sequences and 300 full-length DNA-B sequences was compiled and assembled including their corresponding genomic components from this study using MEGA-11 software. After aligning the DNA-A and DNA-B sequences, the entire assembly was exported in FASTA format for subsequent recombination analysis. GARD and the RDP v5.0 program by Martin et al. (2021) were employed to infer potential recombination events. Within RDP5, seven different algorithms were utilized, and only recombination events and breakpoints supported by at least three distinct algorithms were deemed reliable. Default settings were used for the recombination analysis, with a Bonferroni-corrected p-value of 0.05 set as the cutoff threshold for significance.

### Nucleotide diversity and haplotype variability indices estimation

Full-length genome sequences of TYLCV (38), ToLCPalV DNA-A (69), ToLCPalV DNA-B (42), WmCSV_DNA-A (32), and WmCSV_DNA-B (26) were retrieved from NCBI GenBank database. Out of the analyzed sequences, only these 207 met the inclusion criteria. Sequences with less than 95% coverage of the reference genome, more than 2.5% unknown characters, less than 80% similarity to the reference genome, or redundancy were excluded. All remaining datasets were aligned using the Muscle algorithm within MEGA11 (Tamura et al., 2021). The alignment files were then manually inspected and corrected for any potential inaccuracies.

The calculation of nucleotide diversity (π), denoting the average pairwise number of nt differences per site, was conducted using DnaSP V.5 (Librado and Rozas, 2009). Statistical significance of differences in π between the full viral genomes and their encoded ORFs was assessed by calculating 95% bootstrap confidence intervals. A 100-nt sliding window with a step size of 10 nts was applied across the entire length of virus genome sequences to calculate genetic variation within the nucleotide sequences. Additional parameters related to population genetics, including the number of haplotypes (H), haplotype diversity (Hd), the number of polymorphic sites (S), Watterson’s theta (θw), the total number of mutations (Eta), Tajima’s D (TD) and Fu and Li’s D (FLD), were also calculated for both the full viral genomes and their individual coding regions.

### Nucleotide substitution rate estimation

Nucleotide substitution.site^−1^.year^−1^ (NSSY) and mutation rates were calculated for all begomovirus genomes and their encoded ORFs using the MCMC approach in BEAST (v.1.10.5) with 1×10^8^ chain length (Drummond and Rambaut, 2007). Both strict and relaxed molecular clock models (uncorrelated lognormal) were evaluated for each dataset. Tracer software (Rambaut et al., 2018) was used to analyze BEAST output and assess the best-fitting clock model, mutation rates at the three codon positions of ORFs, and ensure effective sample size (ESS) of at least 200 for all parameters.

### Selection pressure analysis

Selection pressure on the ORFs was assessed through two methods. First, the dN/dS ratio was calculated in MEGA 11. Second, the online web server Datamonkey was employed (www.datamonkey.org) with FUBAR (Fast, Unconstrained Bayesian Approximation) and SLAC (single-likelihood ancestor counting) methods (Weaver et al., 2018).

## Results

### High-throughput sequencing and data analysis

A comprehensive survey conducted across three field plots and two greenhouses in the Al-Hofuf and Qateef municipalities, within the Al-Ahsa province, Saudi Arabia, revealed ~20-30% cucumber plants displaying characteristic begomovirus symptoms of leaf yellowing and the appearance of yellow mosaics (**Figure 1**). Preliminary analyses based on begomovirus core CP amplification, confirmed the presence of begomovirus/es in nine cucumber plants from the Al-Hofuf region. However, no amplification was obtained from samples collected in the Qateef region. The sequenced core CP amplicons displayed the highest nt sequence identities to the DNA-A component of ToLCPalV and WmCSV sequences from the positive cucumber plants. Eight cucumber samples obtained from Al-Hofuf, labeled as 3CuS1, 3CuS2, 4CuS1, 4CuS2, 7CuY1, 7CuY2, 1CuK1, and 1CuK2, were selected for Illumina high-throughput sequencing based on initial detection. From these samples, eight DNA libraries were generated, producing 2,349,152, 2,260,726, 2,765,232, 2,782,626, 2,895,278, 2,980,606, 2,599,258, and 2,452,699 raw paired reads, respectively. Through BLASTn analysis of assembled contigs in the NCBI GenBank database, we successfully identified candidate bipartite and monopartite begomovirus genomes. However, no DNA-satellites could be identified. The full-length genomic sequences have been deposited in the NCBI GenBank database, and each sequence has been assigned an accession number for future reference (**Supplementary Table 1**).

### Deciphering begomovirus genomes through comparative sequence analysis

**Figure 2:**
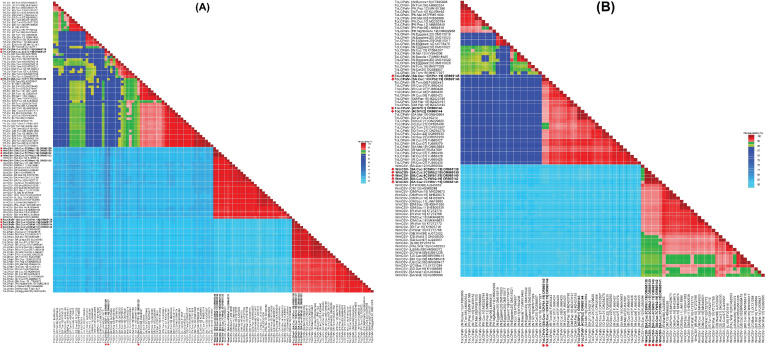
A matrix for species demarcation was constructed by evaluating pairwise nucleotide sequence identities of: **(A)** full-length genomes of identified viruses tomato yellow leaf curl virus (TYLCV), tomato leaf curl Palampur virus (ToLCPalV) DNA-A, and watermelon chlorotic stunt virus (WmCSV) DNA-A, **(B)** ToLCPalV DNA-B and WmCSV DNA-B genomic sequences. The species demarcation tool (SDT v. 1.2) facilitated this analysis. To visually represent the results, a color-coded matrix was generated, employing three distinct shades: red, green, and blue. These colors were utilized to define species and strain demarcation thresholds for begomovirus genomic components, aligning with the criteria outlined by Muhire et al. (2013).

**Figure 3:**
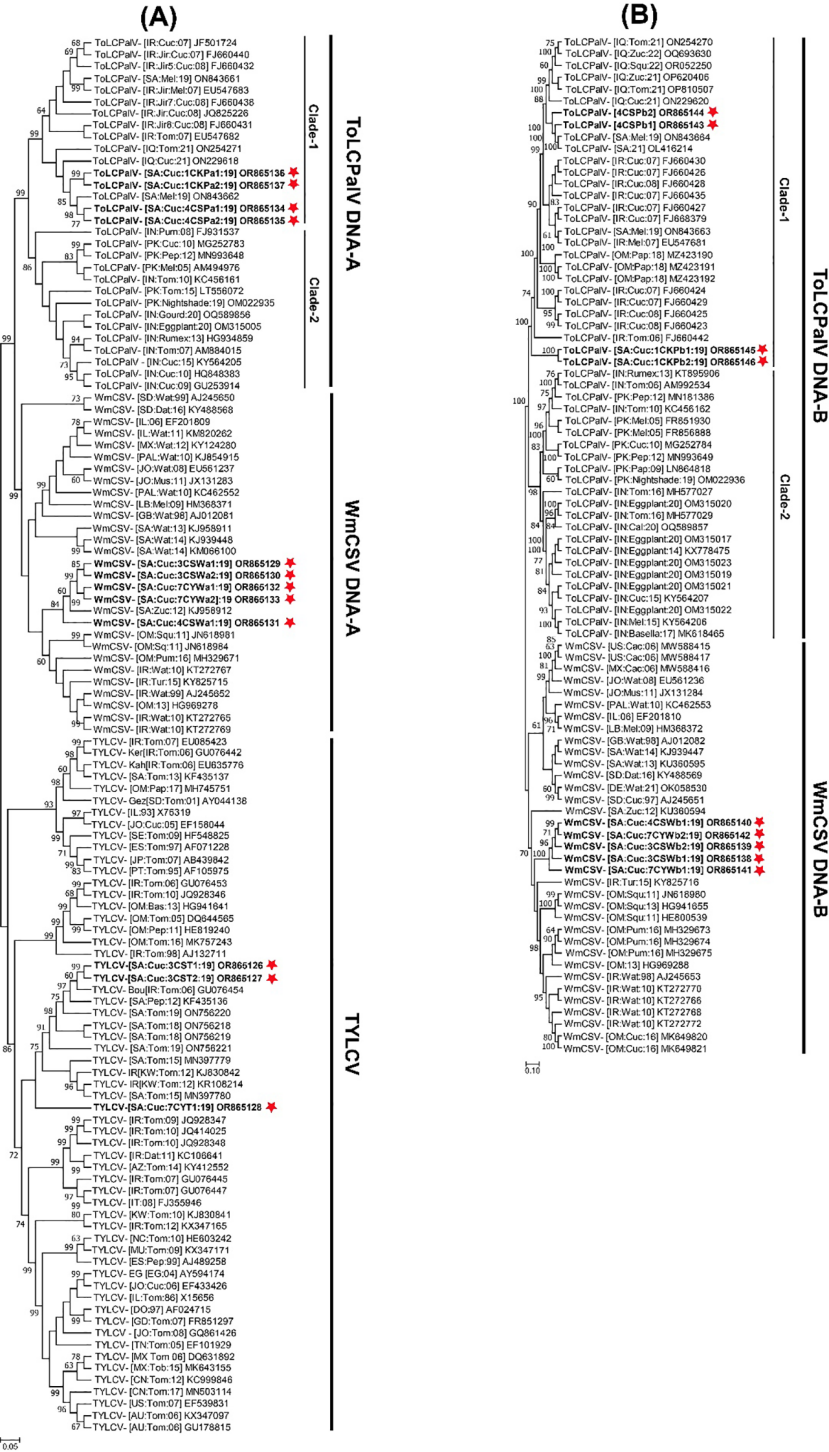
Phylogenetic dendrograms, constructed using the maximum likelihood method, illustrate the evolutionary relationships of **(A)** full-length genomes of identified tomato yellow leaf curl virus (TYLCV), tomato leaf curl Palampur virus (ToLCPalV) DNA-A, watermelon chlorotic stunt virus (WmCSV) DNA-A and **(B)** ToLCPalV DNA-B, WmCSV DNA-B. For comparison, full-length sequences of the most closely related begomovirus genomes were obtained from the NCBI GenBank database. Each isolate’s host plant species, geographical origin, and database accession numbers are provided in the tree, with the begomovirus isolates from this study highlighted in bold and marked with a red star. The trees were constructed with 1000 replicates for percentage bootstrap values. Taxonomic abbreviations for begomovirus species adhere to the guidelines outlined by Zerbini et al. (2017).

Pairwise nt sequence identity analysis using SDT indicated that the cucumber samples harbored either a single begomovirus genome or a combination of multiple begomoviruses. Sequences 3CST1 and 3CST2 exhibited a 98.2% identity to each other, with their highest nt sequence identities at 95.9% and 94.7%, respectively, matching the TYLCV isolate (GU076454) of “Boushehr” strain reported from Iran (**Figure 2A**) (Lefeuvre et al., 2010). Sequence 7CYT1 showed 90-91.2% identity to 3CST1 and 3CST2 isolates, sharing its highest nt sequence identity at 94.6% with TYLCV isolate (GU076447) reported from Iran (Lefeuvre et al., 2010). In the phylogenetic dendrogram, isolates 3CST1 and 3CST2 formed a well-supported clade (100% bootstrap value) with other isolates of Boushehr and Iran strains of TYLCV from Saudi Arabia, Kuwait, and Iran (**Figure 3A**). However, isolate 7CYT1 constituted a monophyletic clade in the phylogenetic dendrogram.

Sequences 3CSWa1, 3CSWa2, 4CSWa1, 7CYWa1, and 7CYWa2 shared mutual nt sequence identities of 98-100%, with their highest nt sequence identities of 97.9-98.7% aligning with the DNA-A component of a WmCSV isolate (KJ958912) reported from squash plants in Saudi Arabia (**Figure 2A**) (Rezk et al., 2019). In the phylogenetic dendrogram, these isolates formed a well-supported clade (100% bootstrap value) separate from other WmCSV isolates, with WmCSV (KJ958912). Sequences 3CSWb1, 3CSWb2, 4CSWb1, 7CYWb1, and 7CYWb2 exhibited 99.3-100% mutual nt sequence identities, sharing their highest identities of 95.8-96.3% with the DNA-B component of a WmCSV isolate (AJ245653) reported from Iran infecting watermelon crops (**Figure 2B**) (Kheyr-Pour et al., 2000). Our analysis revealed that these isolates formed a strongly supported clade (100% bootstrap value) distinct from other WmCSV isolates, as shown in **Figure 3B**.

Sequences 4CSPa1, 4CSPa2, 1CKPa1, and 1CKPa2 were 99.4-100% identical to each other, with their highest nt sequence identities at 99.1-99.6% aligning with the DNA-A of a ToLCPalV isolate (ON843662) previously reported infecting melon crop in Saudi Arabia (**Figure 2A**) (Alhudaib et al., 2014). The phylogenetic dendrogam grouped these isolates with other ToLCPalV isolates reported from Saudi Arabia and Iraq into a separate well-supported clade (**Figure 3A**). Sequences 4CSPb1 and 4CSPb2 exhibited 100% mutual nt sequence identity, sharing their 100% nt sequence identity with the DNA-B of ToLCPalV isolate (ON843664) infecting melon crop in Saudi Arabia (Alhudaib et al., 2014). Conversely, sequences 1CKPb1, and 1CKPb2 were 100% identical to each other, with their highest nt sequence identity (96.2%) aligning with the DNA-B of a ToLCPalV isolate (FJ660442) reported from tomato crop in Iran (**Figure 2B**) (Heydarnejad et al., 2009). However, these were only 94.1% identical to 4CSPb1 and 4CSPb2 isolates. In the phylogenetic dendrogram, isolates 4CSPb1 and 4CSPb2 grouped well with ToLCPalV isolate (ON843664) within a well-supported clade (100% bootstrap value) with other ToLCPalV isolates reported from Iran and Iraq (**Figure 3B**). Meanwhile, isolates1CKPb1 and 1CKPb2 formed a separate well-supported (100% bootstrap value) monophyletic clade.

The presence of begomovirus components in the corresponding cucumber plant samples was further confirmed using specifically designed primers for each component (**Supplementary Table 2**).

### Exploring potential recombination events

**Figure 4:**
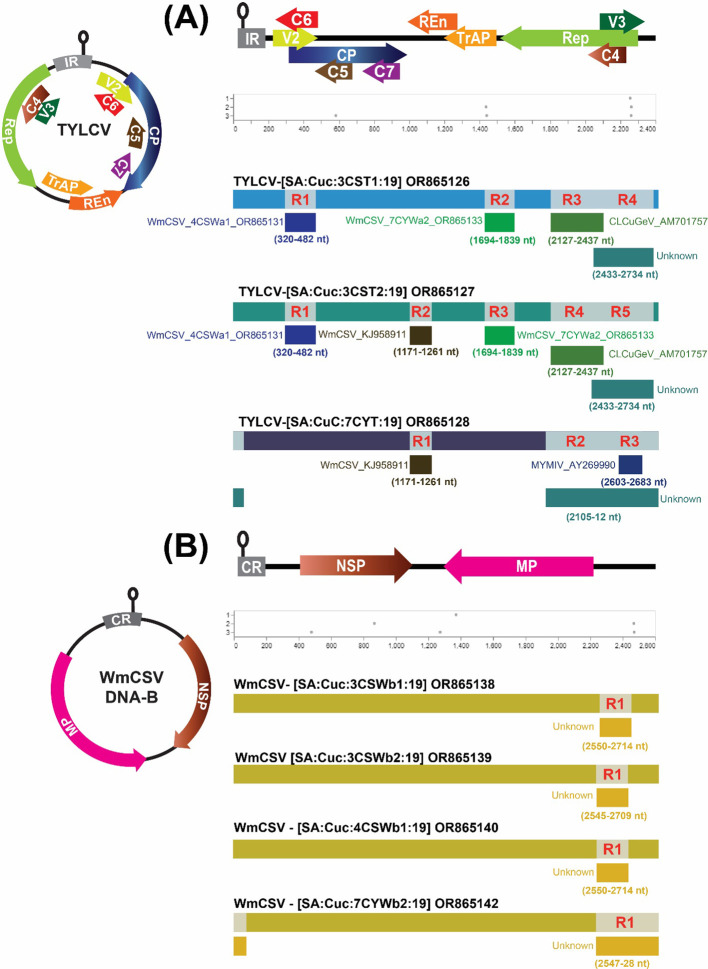
Identification of recombination events through the implementation of GARD and Recombination Detection Program (RDP5.0). Panel **(A)** illustrates the recombination analysis for potentially recombinant TYLCV, while Panel **(B)** depicts the same analysis for WmCSV DNA-B isolates. The graphical representation of TYLCV and WmCSV DNA-B is succeeded by GARD and RDP analyses, respectively.

Initially, the automated GARD tool within Datamonkey was employed to search for possible signs of recombination within the genomic sequences of the identified begomoviruses. The analysis conducted by GARD revealed both robust and subtle recombination signals present in the DNA-A and DNA-B genomic regions. Subsequent data analysis using the RDP method indicated that four potential recombination breakpoints were exclusively detected in the TYLCV isolates 3CST1, five in 3CST2 and three in 7CYT1, respectively (**Figure 4A**). The major parents in these recombination events were different TYLCV isolates, whereas the minor parents were isolates of different begomoviruses, namely WmCSV, CLCuGeV, TYLCV and MYMIV (**Supplementary Table 3**).

Only one recombination event was detected in the DNA-B of WmCSV isolates 3CSWb1, 3CSWb2, 4CSWb1 and 7CYWb2 (**Figure 4B**; **Supplementary Table 3**). Potentially, the DNA-B of WmCSV isolate (KY825716) from Iran and an unknown DNA-B isolate were detected as the major and minor parents for the DNA-B of WmCSV isolates. No notable recombination events were observed in the DNA-A or DNA-B components of other begomovirus genomes.

### Estimating genetic variation within the identified begomovirus populations

The study investigated the dynamics of sequence variations within TYLCV, WmCSV, and ToLCPalV populations. Among the begomoviruses and their genomic components, ToLCPalV DNA-B exhibited the highest S and Eta values, with 1182 and 1588, respectively. In contrast, WmCSV DNA-A had the least S and Eta values, with 369 and 426, respectively. These S and Eta values were also determined separately for individual ORFs of each begomovirus genomic component (**Supplementary Table 4**). The AC1ToLCPalV (553 and 767), AC1WmCSV (126 and 148) and C1TYLCV (431 and 536) demonstrated the highest S and Eta values, while, the AC4WmCSV and AC4ToLCPalV, and V2TYLCV exhibited the lowest values. The DNA-B of ToLCPalV showed the highest number of inDels in the entire begomovirus population dataset, whereas among the ORF dataset, the C1TYLCV had the highest number of inDels (30). The DNA-B of also had the highest number of pairwise nt differences (k) in comparison to the entire population dataset (278.3), while the C1TYLCV produced the highest k values (110.71) among the ORF dataset.

**Figure 5:**
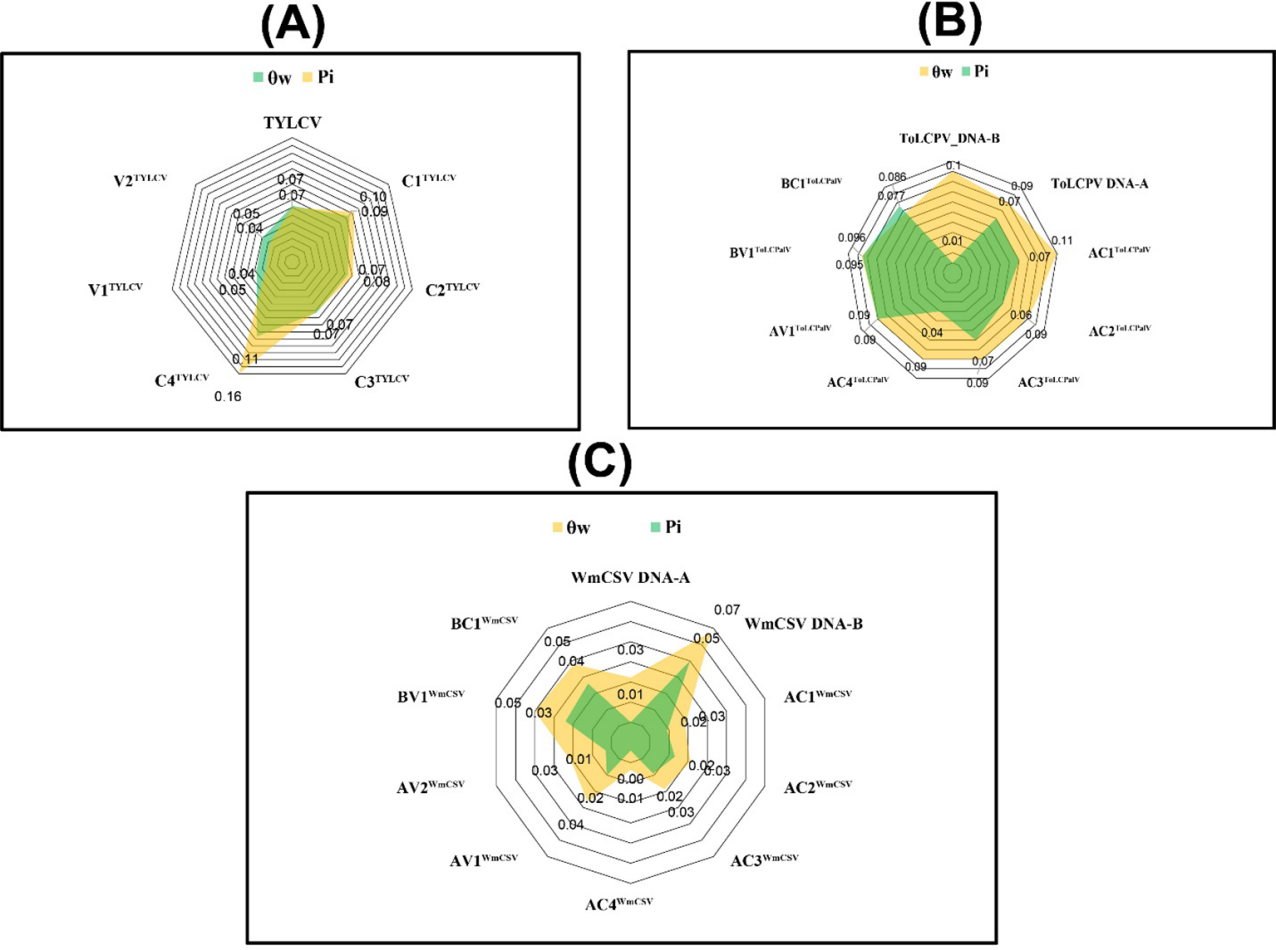
Calculation of genetic diversity metrics, including Pi (π) and Watterson’s theta (θw), for **(A)** TYLCV, **(B)** ToLCPalV (DNA-A and DNA-B), **(C)** WmCSV (DNA-A and DNA-B), and their respective open reading frames (ORFs).

The average nt diversity within the population dataset indicated identical π values (0.07) for TYLCV and the DNA-A of ToLCPalV (**Figure 5**; **Supplementary Table 4**). Within the ORF dataset, the C4TYLCV displayed the highest π value (0.160). Additionally, the θw value was highest (0.102) in the DNA-B of ToLCPalV compared to the entire population dataset (**Figure 5**). However, the C4TYLCV and AC1ToLCPalV exhibited a higher number of segregating sites with their higher θw values (0.107 and 0.106), respectively.

Across all population datasets, neutrality indices calculated through TD and FLD tests were negative (**Supplementary Table 4**). The DNA-A of WmCSV showed highly negative TD and FLD values (-1.74 and -2.53, respectively). Similarly, the neutrality indices of the ORF dataset were negative for all ORFs studied, except for the C4TYLCV. Among the remaining ORFs, the AC4ToLCPalV displayed highly negative TD and FLD values (-2.42 and -4.79, respectively). Notably, TYLCV and the DNA-B of ToLCPalV had the lowest FLD values (-0.70 and -0.24), suggesting that natural selection may play a minimal role in their diversification.

### Selection pressure and estimation of evolutionary rate for individual ORFs of begomovirus components

The Fast, Unconstrained Bayesian Approximation (FUBAR), Single Likelihood Ancestor Counting (SLAC), and dN/dS substitution rate ratios were employed to assess the presence of negative selection pressure acting on each ORF encoded by the identified begomoviruses. According to FUBAR results, the AC2^ToLCPalV^ had the most positive sites (6), followed C2^TYLCV^ and C3^TYLCV^ with 5 positive sites, respectively (**Supplementary Table 5**). Interestingly, in the recent SLAC analysis, most ORF sites exhibited minimal evidence of positive selection. Conversely, the same analysis showed that these sites were predominantly under negative selection. FUBAR and SLAC tests identified 156 and 104 sites under negative selection within the AC1^ToLCPalV^, followed by the C1^TYLCV^ (with 130 and 59 sites) and AC1^WmCSV^ (with 123 and 90 sites), respectively.

The average dN/dS ratio of all ORFs was recorded to be less than 1, except for the C4^TYLCV^, AC4^WmCSV^ and AC4^ToLCPalV^ with dN/dS ratios of 1.72, 1.67 and 1.19, respectively (**Supplementary Table 5**). The lowest dN/dS ratio was observed in the AV1^ToLCPalV^ (0.02) and V1^TYLCV^ (0.09), respectively. The observed variations within these ORFs suggest they have undergone natural or purifying selection pressure.

Utilizing the 95% high-probability density (HPD) interval, we statistically assessed the rate estimates and the mean value of nt substitutions, comparing them under both strict and relaxed uncorrelated molecular clock models for all begomovirus genomic components (**Supplementary Table 6**) and their corresponding ORFs (**Supplementary Table 7**). The estimates varied significantly across all datasets. Among the begomovirus populations, the rates were highest in TYLCV (6.28E-2, 14.82E-2) and lowest for WmCSV DNA-A (1.87E-2, 4.38E-2) (**Supplementary Table 6**). Meanwhile, among the ORF dataset, the AV1^ToLCPalV^ had the highest (33.46-118.07E-2) and the AV1^WmCSV^ had the lowest (1.18-21.38E-2) substitution site^-1^. year^-1^ (**Supplementary Table 7**).

We further analyzed the impact of nt substitutions on the three codon positions (CoP) of all ORFs under both stringent and relaxed evolutionary rate models. Among all ORFs, the AC2^ToLCPalV^ exhibited a higher mutation rate at the first codon position (CoP1), while C2^TYLCV^ showed the highest rate at CoP2. All remaining ORFs displayed a higher mutation rate at CoP3 (**Supplementary Table 7**).

## Discussion

In Saudi Arabia, small farms utilize diverse cultivation methods, including traditional open-field cropping and controlled greenhouse environments, for cucumber production (Mousa et al., 2019). Research conducted in the Western region has identified the presence of both ToLCSDV and tomato yellow leaf curl betasatellite (TYLCB) within these agricultural ecosystems (Sohrab et al., 2017). A paucity of research on begomovirus molecular characterization in Saudi Arabia has left a knowledge gap regarding economically important geminiviruses (Rezk et al., 2019; Sohrab, 2020). In a prior investigation employing a high-throughput sequencing approach, we successfully identified mixed infections of CLCuGeV associated with DNA-satellites and/or TYLCV and ToLCPalV infecting tomato and muskmelon crop plants in Saudi Arabian fields (AlHudaib et al., 2022).

The current survey in the Eastern region of Saudi Arabia, identified cucumber plants with characteristic begomovirus symptoms, including leaf yellowing and yellow mosaics. Initial analyses confirmed the presence of begomovirus/es in nine symptomatic cucumber plants from the Al-Hofuf region using begomovirus core CP amplification. However, no amplification was obtained from samples in the Qateef region. The subsequent high-throughput sequencing of eight selected cucumber samples from Al-Hofuf revealed diverse begomovirus sequences, with full-length sequences identified in each sample. Comparative sequence analysis, phylogenetic dendrograms and recombination analysis unveiled that cucumber samples were infected with either a single begomovirus genome or a mixture of multiple begomoviruses involving TYLCV, ToLCPalV and WmCSV. The phylogenetic dendrogram, shows that TYLCV isolates formed a clade with Boushehr and Iran strains of other TYLCV isolates from Saudi Arabia, Kuwait, and Iran. Nevertheless, TYLCV isolate 7CYT1 constituted a distinct monophyletic clade, separate from other TYLCV isolates (**Figure 3**). These results suggest the possibility that TYLCV isolates may have originated from two independent introductions into Saudi Arabia.

The WmCSV isolates showed high mutual nt sequence identities, aligning with the DNA-A component of WmCSV isolate reported from squash plants in Saudi Arabia (Rezk et al., 2019). In recent years, the emergence of two begomoviruses has posed a significant threat to cucurbit cultivation in the Middle East. Specifically in cucurbits Lapidot et al. (2014) documented the invasion of a NW begomovirus, squash leaf curl virus (SLCV), and an OW begomovirus, WmCSV. The phylogenetic dendrograms in the current study revealed distinct clades for DNA-A and DNA-B sequences of WmCSV isolates from Saudi Arabia separate from other WmCSV isolates, suggesting its successful establishment in the region, after its introduction. Previous reports of WmCSV from cucurbit crops support this speculation (Al-Saleh et al., 2014; Sattar, 2018; Rezk et al., 2019). It is also evident that WmCSV has now been spreading into the cucurbit fields in Saudi Arabia. This unique evolutionary trajectory indicates that WmCSV has been adapted and diversified within Saudi Arabia, highlighting its potential for long-term persistence in this region.

The DNA-A of ToLCPalV isolates exhibited high nt sequence identities with ToLCPalV isolates reported from Saudi Arabia and Iran. These isolates, along with other ToLCPalV isolates from Saudi Arabia, formed a distinct clade in the phylogenetic dendrogram. Conversely, the DNA-B isolates displayed varied nt sequence identity with the DNA-B of ToLCPalV isolates from Saudi Arabia and Iran. Furthermore, these isolates segregate into two independent clusters in the phylogenetic dendrogram. Isolates 4CSPb1 and 4CSPb2 clustered with ToLCPalV DNA-B from Saudi Arabia, Iran and Iraq, while isolates 1CKPb1 and 1CKPb2 formed a distinct clade (**Figure 3**). ToLCPalV, a bipartite begomovirus, is extensively distributed in India and Pakistan, where it has been documented infecting various host plants such as tomato, cucurbits (Ali et al., 2010; Namrata et al., 2010; Shafiq et al., 2019; Hanamasagar et al., 2021), and more recently, a weed (Sattar et al., 2022). It was first reported in 2009 from Iran (Fazeli et al., 2009), where it has been identified infecting common bean, cucumber, melon, pumpkin, and watermelon crops (Heydarnejad et al., 2009, 2013; Esmaeili et al., 2015). The discovery of ToLCPalV in Saudi Arabia suggests a wider geographic distribution than previously understood. A detailed analysis revealed that the ToLCPalV isolates prevailing in the Arabian Peninsula differ significantly from those in Southeast Asia (**Figure 3**). Both groups are separate in the phylogenetic dendrogram, suggesting their independent evolution from a common ancestor and parallel establishment in different niches. It is more probable that the introduction of these viruses into the Saudi Arabian agroecosystem originated from Iran.

In this current investigation, the complete genomes of TYLCV, ToLCPalV and WmCSV were assessed for genomic variations present in their respective genetic compositions. Surprisingly, our findings revealed that the analyzed begomoviruses primarily undergo evolution influenced by strong negative pressure. Notably, no instances of recombination were observed in the DNA-A components, except for TYLCV isolates and the DNA-B of WmCSV. The TYLCV, ToLCPalV, and WmCSV begomovirus populations exhibited a high level of genetic diversity indices, with ToLCPalV DNA-B displaying the highest genetic diversity among all the genomic components of the studied begomoviruses. In contrast, WmCSV DNA-A displayed the lowest genetic diversity indices, indicating that low level of genetic differentiation is operating in this population. Furthermore, within the examined datasets of begomovirus populations, the mean number of segregating sites (θw) and the negative TD values consistently suggest that these populations are undergoing diversification to different extents, primarily driven by purifying selection. Another possible notion is that these begomovirus populations may have recently expanded, as opposed to undergoing neutral selection (Srivastava et al., 2022). The presence of low-frequency alleles within these populations could potentially account for the notably negative TD and FLD values (Farooq et al., 2021). The genetic variations (π and θw) observed in the populations of TYLCV and ToLCPalV surpass those found in the WmCSV population. These results highlight significant sequence divergence and a greater prevalence of unique mutations within the TYLCV and ToLCPalV populations, underscoring their pivotal roles in their emergence. Despite the prevailing notion that DNA-A components predominantly contribute to begomovirus evolution, some recent studies suggest that DNA-B can also play a pivotal role in begomovirus evolution (Sattar et al., 2022). Our findings revealed remarkable genetic diversity indices in the DNA-B of ToLCPalV and WmCSV compared to their DNA-A counterparts, supporting the speculation that DNA-B may actively assist the virus in adapting swiftly, particularly in response to pronounced negative pressure.

Our findings regarding the ORFs encoded by begomoviruses revealed distinct genetic diversity indices for each ORF, highlighting the non-uniform distribution of nt diversity and genetic mutations across their sequences. Specifically, AC1 (C1 or Rep) demonstrated the highest values across various genetic diversity indices, whereas AV2 (V2) exhibited the lowest values in all datasets. Previous studies have consistently reported higher nt variability in the Rep genes of begomoviruses (Lima et al., 2017). Our findings further support the non-random distribution pattern of nt diversity and genetic mutations observed within the ORFs of various begomoviruses. Furthermore, when comparing populations ORFs using k values, it was observed that the Rep consistently exhibited the highest k values across all ORF datasets. This underscores a higher degree of genetic differentiation. In contrast, AC4 and V2 displayed a lower level of genetic differentiation. The consistent occurrence of negative values for both TD and FLD across all begomovirus populations and their corresponding ORFs highlights the conserved nature of their genes. These findings are likely in populations where predominant nt variations are transient and subsequently eradicated by purifying selection (Mishra et al., 2022). Furthermore, varied dN/dS ratios observed in distinct begomovirus-encoded ORFs suggest the influence of diversifying selection. Notably, the elevated dN/dS ratio observed in AC4 (C4) implies that this particular gene is undergoing evolution under rigorous purifying and negative selection. Similar observations have been previously identified in various viruses (Aguilar Rangel et al., 2023), as well as in different begomoviruses such as ChiLCV (Mishra et al., 2022) and ToLCPalV (Sattar et al., 2022). In our investigation, the majority of begomovirus components exhibited elevated NSSY rates. This aligns with previous research indicating that geminiviruses consistently demonstrate high NSSY rates, similar to many RNA viruses (Duffy and Holmes, 2009). While we employed both strict and relaxed molecular clocks to estimate the NSSY rate, the strict molecular clock produced more favorable results. This finding contrasts with a previous study that leaned towards the relaxed molecular clock (Srivastava et al., 2022).

The analysis of recombination revealed prevalent recombinants within the populations of TYLCV and WmCSV DNA-B. The presence of both intra- and interspecies recombination events was indicated by the identification of unknown recombinant parents, as well as parents from different species. The presence of well-supported recombination events in TYLCV isolates, involving multiple begomoviruses as major and minor parents, further strengthens the hypothesis that TYLCV has been introduced into Saudi Arabia on multiple occasions. High mutation rate and strong purifying selection likely drove the independent evolution of ToLCPalV in the absence of recombination, as evidenced by the nucleotide diversity indices and recombination analysis. This speculation is further supported by a study conducted by Sattar et al. (2022). Our study detected four instances of mixed infections in plants, where TYLCV and ToLCPalV co-occurred alongside WmCSV isolates. Dual and even triple begomovirus infections within single plants are well-documented (Leke et al., 2011; Charoenvilaisiri et al., 2020; Batista et al., 2022; Rahman et al., 2023). These co-infections potentially contribute to the emergence of novel recombinant or pseudo-recombinant begomoviruses. This, coupled with a large vector population, can significantly enhance intra-regional genetic exchange among begomoviruses (Ouattara et al., 2022). Geminiviruses replicate through a rolling-circle mechanism, where the Rep protein binds to specific iteron sequences. In bipartite begomoviruses, similar iteron sequences in DNA-A and DNA-B facilitate successful replication (Hanley-Bowdoin et al., 2013). This explains why, during mixed infections involving monopartite and bipartite begomoviruses, the DNA-B component might not be efficiently trans-replicated by the monopartite begomovirus genome (Ouattara et al., 2022).

Overall, the comprehensive analysis of high-throughput sequencing data and comparative sequence analysis sheds light on the diversity and coexistence of begomovirus genomes in cucumber crops in Al-Ahsa, Saudi Arabia. The identified strains and isolates contribute valuable information to the understanding of begomovirus dynamics in the region.

## Conclusion

Our study provides insights into the begomovirus dynamics in cucumber crops in Al-Ahsa, Saudi Arabia. The introduction of diverse begomoviruses into the Saudi Arabian agroecosystem raises concerns for agriculture, with potential factors including extensive trade, cross-border travel, and various transportation means contributing to the overland introduction of these viruses. The study revealed that TYLCV and ToLCPalV isolates originated from multiple introductions into Saudi Arabia. The begomovirus populations in our study underwent evolution under strong purifying selection and low recombination rate. Additionally, mixed infections involving TYLCV, ToLCPalV, and WmCSV in the same plants may contribute to the emergence of novel recombinants. This study contribute valuable information for future research, disease management, and the development of strategies to mitigate the impact of begomoviruses on agriculture in the Middle East.

## Data Availability

The datasets presented in this study can be found in online repositories. The names of the repository/repositories and accession number(s) can be found below: GenBank, OR865126-OR865146.
