# Supplementary material for: High-throughput sequencing discovered diverse monopartite and bipartite begomoviruses infecting cucumbers in Saudi Arabia
Source: Front Plant Sci. 2024 Oct 10;15:1375405. doi: 10.3389/fpls.2024.1375405 (PMC11499130; doi:10.3389/fpls.2024.1375405)
Supplement: Supplementary file 1 [file Table1.docx]

Supplementary Material

# Supplementary Data

**Supplementary Table 1:** Collection of samples and whole genome sequencing using the Illumina MiSeq platform

| **Plant** | **Sample** | **Place** | **TYLCV** | **WmCSV DNA-A** | **WmCSV DNA-B** | **ToLCPalV DNA-A** | **ToLCPalV DNA-B** |
| --- | --- | --- | --- | --- | --- | --- | --- |
| Cucumber (*Cucumis sativus* L.) | 3CuS1 | F1**^*^** | 3CST1 (OR865126) | 3CSWa1 (OR865129) | 3CSWb1 (OR865138) | - | - |
|  | 3CuS2 |  | 3CST2 (OR865127) | 3CSWa2 (OR865130) | 3CSWb2 (OR865139) | - | - |
|  | 4CuS1 | F2**^**^** | - | 4CSWa1 (OR865131) | 4CSWb1 (OR865140) | 4CSPa1 (OR865134) | 4CSPb1 (OR865143) |
|  | 4CuS2 |  | - | - | - | 4CSPa2 (OR865135) | 4CSPb2 (OR865144) |
|  | 7CuY1 | G1**^***^** | 7CYT1 (OR865128) | 7CYWa1 (OR865132) | 7CYWb1 (OR865141) | - | - |
|  | 7CuY2 |  | - | 7CYWa2 (OR865133) | 7CYWb2 (OR865142) | - | - |
|  | 1CuK1 | G2**^****^** | - | - | - | 1CKPa1 (OR865136) | 1CKPb1 (OR865145) |
|  | 1CuK2 |  | - | - | - | 1CKPa2 (OR865137) | 1CKPb2 (OR865146) |

**^*^** F1: Farm number 1 (Al-Hussain farm) at the Date Palm Research Station, King Faisal University, Al- Hofuf

**^**^** F2: Farm number 2 at the Date Palm Research Station, King Faisal University, Al-Hofuf

**^***^** G1: Greenhouse at Yaseen Vegetable Farm, Al- Hofuf

**^****^** G2: Greenhouse at College of Agriculture, King Faisal University, Al- Hofuf

**Supplementary Table 2:** Primer sequences employed in the present study

| **Primers** | **Primer sequence** | **Nucleotide position** | **PCR product** | **Reference** |
| --- | --- | --- | --- | --- |
| AC1048 | GGRTTDGARGCATGHGTACATG |  | Core coat protein | (Wyatt and Brown, 1996) |
| AV494 | GCCYATRTAYAGRAAGCCMAG |  |  |  |
| TY-Cp_F | TGAAGGCCCATGTAAAGTCCAG | 495-516 | TYLCV | (AlHudaib et al., 2022) |
| TY-Cp_R | CATAGAAATAGATACGTATTTTC | 1026-1048 |  |  |
| PA-Cp_F | AGCTCTGACGTGCCCAGGGGCT | 460-481 | ToLCPalV DNA-A |  |
| PA-Cp_R | CACCGAATCGTAAAAATAGATC | 1020-1041 |  |  |
| PB-C1C_F | CGTTTGTGAGCGCGTACTCAATAC | 2062-2085 | ToLCPalV DNA-B |  |
| PB-C1C_R | AATATTATATACGAAAGGCCCCTT | 2697-2720 |  |  |
| Wm-AV1_F | CGAAGCGAACAGGAGATATTCTC | 318-341 | WmCSV DNA-A | This study |
| Wm-AV1_R | CAATGTAGCATACACAGGATTCG | 1029-1052 |  |  |
| Wm-BV1_F | ACCAGTGATGCGTCGCTATGATG | 493-515 | WmCSV DNA-B |  |
| Wm-BV1_R | TTCAGCCCACATAATCCAGTAC | 1235-1256 |  |  |

**Supplementary Table 3:** Detection of potential breakpoints indicative of recombination using seven distinct approaches in RDP5

| **Recombinant** | **Event No.** | **Breakpoints^*^** | | **Parents^**^** | | **Methods^***^** | **p-value** |
| --- | --- | --- | --- | --- | --- | --- | --- |
|  |  | **Begin** | **End** | **Major** | **Minor** |  |  |
| TYLCV_3CST1 (OR865126) | R1 | 320 | 482 | TYLCV_ON756220 (94.2) | WmCSV_4CSWa1 (74.5) | ***R,G,B,M,C,S,3S*** | 3.98E-19 |
|  | R2 | 1694 | 1839 | TYLCV_7CYT1 (90.0) | WmCSV_7CYWa2 (75.4) | ***R,G,****C,S****,3S*** | 2.73E-13 |
|  | R3 | 2127 | 2437 | TYLCV_ON756221 (91.3) | CLCuGeV_AM701757 (76.7) | ***R,G,B,M,C,S,3S*** | 8.96E-29 |
|  | R4 | 2433 | 2734 | TYLCV_GU076442 (91.9) | Unknown (TYLCV_AY044138) (89.1) | ***R,G,M,C,S,3S*** | 9.73E-09 |
| TYLCV_3CST2 (OR865127) | R1 | 320 | 482 | TYLCV_ON756220 (94.7) | WmCSV_4CSWa1 (75.0) | ***R,G,B,M,C,S,3S*** | 3.98E-19 |
|  | R2 | 1171 | 1261 | TYLCV_FJ355946 | WmCSV_KJ958911 (74.4) | ***R,G,3S*** | 5.91E-10 |
|  | R3 | 1694 | 1839 | TYLCV_7CYT1 (91.2) | WmCSV_7CYWa2 (76.0) | ***R,G,****C,S****,3S*** | 2.73E-13 |
|  | R4 | 2127 | 2437 | TYLCV_ON756221 (90.5) | CLCuGeV_AM701757(76.8) | ***R,G,B,M,C,S,3S*** | 8.96E-29 |
|  | R5 | 2418 | 2734 | TYLCV_GU076442 (91.4) | Unknown (TYLCV_AY044138) (88.8) | ***R,G,M,C,S,3S*** | 9.73E-09 |
| TYLCV_7CYT1 (OR865128) | R1 | 1189 | 1279 | TYLCV_FJ355946 (94.0) | WmCSV_KJ958911 (72.6) | ***R,G,3S*** | 5.91E-10 |
|  | R2 | 2105 | 12 | TYLCV_X76319 (88.6) | Unknown (TYLCV_JQ414025) (94.1) | ***R,G,B,M,C,S,3S*** | 8.85E-54 |
|  | R3 | 2603 | 2683 | TYLCV_AY044138 (87.2) | MYMIV_AY269990 | ***R,G,M,****C,S****,3S*** | 9.97E-10 |
| WmCSV_DNA-B_3CSWb1 (OR865138) | R1 | 2550 | 2714 | WmCSV_DNA-B_KY825716 (94.9) | Unknown (WmCSV_DNA-B _MH329673) (95.0) | ***R,G,B,****M****,C,3S*** | 1.46E-39 |
| WmCSV_DNA-B_3CSWb2 (OR865139) | R1 | 2545 | 2709 | WmCSV_DNA-B _KY825716 (94.9) | Unknown (WmCSV_DNA-B _MH329673) (95.0) | ***R,G,B,****M****,C,3S*** | 1.46E-39 |
| WmCSV_DNA-B_4CSWb1 (OR865140) | R1 | 2550 | 2714 | WmCSV_DNA-B_KY825716 (94.9) | Unknown (WmCSV_DNA-B _MH329673) (94.9) | ***R,G,B,****M****,C,3S*** | 1.46E-39 |
| WmCSV_DNA-B_7CYWb2 (OR865142) | R1 | 2547 | 28 | WmCSV_DNA-B_KY825716 (94.9) | Unknown (WmCSV_DNA-B _MH329673) (95.5) | ***R,G,B,****M****,C,3S*** | 1.46E-39 |

Abbreviations: 3S, Sequence Triplets; B, Bootscan; C, Chimarea; G, Geneconv; M, MaxChi; R, RDP; S, SiScan.

* Each breakpoint is denoted by the nucleotide position on the corresponding reference genome.

** The percentages in parentheses signify the nucleotide sequence identity of each recombinant isolate with the predominant begomovirus species as the major and minor parent.

*** Methods with cut-off p-values > 0.05 are highlighted in bold and italicized text, while the method displaying the highest p-value is underlined and presented in the respective column.

**Supplementary Table 4:** Genetic diversity metrics for TYLCV, ToLCPalV (DNA-A and DNA-B), and WmCSV (DNA-A and DNA-B), along with assessments of their individual ORFs

| **Datasets** | **Number seq** | **InDel sites** | **S** | **Eta (h)** | **Number of variants** | **Hd** | **Pi (π)** | **k** | **h** | **θ_w_** | **Neutrality test** | |
| --- | --- | --- | --- | --- | --- | --- | --- | --- | --- | --- | --- | --- |
|  |  |  |  |  |  |  |  |  |  |  | **TD** | **FLD** |
| TYLCV | 41` | 49 | 786 | 958 | 408/9/14 | 1 | 0.07 | 192.01 | 41 | 0.072 | -0.53 | -0.70 |
| ToLCPalV DNA-A | 73 | 55 | 1141 | 1554 | 453/239/66 | 0.998 | 0.07 | 208.2 | 69 | 0.087 | -1.22 | -2.17 |
| ToLCPalV_DNAB | 46 | 89 | 1182 | 1588 | 614/248/68 | 0.996 | 0.01 | 278.3 | 43 | 0.101 | -0.85 | -0.24 |
| WmCSV_DNA-A | 37 | 30 | 369 | 426` | 138/33/4 | 0.997 | 0.01 | 54.4 | 35 | 0.032 | -1.74 | -2.53 |
| WmCSV DNA-B | 31 | 84 | 732 | 881 | 313/96/14 | 0.996 | 0.05 | 138.08 | 29 | 0.068 | -1.45 | -1.53 |
| C1^TYLCV^ | 41 | 30 | 431 | 536 | 211/83/8 | 1 | 0.103 | 110.71 | 41 | 0.094 | -0.43 | -0.66 |
| C2^TYLCV^ | 41 | 0 | 129 | 152 | 66/17/2 | 0.984 | 0.081 | 33.04 | 32 | 0.074 | -0.25 | -1.28 |
| C3^TYLCV^ | 41 | 1 | 125 | 153 | 59/20/2 | 0.927 | 0.071 | 28.9 | 31 | 0.073 | -0.69 | -1.43 |
| C4^TYLCV^ | 41 | 0 | 139 | 174 | 89/30/2 | 0.971 | 0.160 | 48.03 | 32 | 0.107 | 0.66 | 0.73 |
| V1^TYLCV^ | 41 | 0 | 158 | 177 | 85/13/2 | 0.991 | 0.035 | 27.43 | 35 | 0.047 | -1.22 | -1.26 |
| V2^TYLCV^ | 41` | 0 | 73 | 80 | 52/5/1 | 0.965 | 0.038 | 13.16 | 26 | 0.049 | -1.07 | -0.19 |
| AC1^ToLCPalV^ | 73 | 16 | 553 | 767 | 220/107/38 | 0.996 | 0.074 | 80.25 | 64 | 0.105 | -1.72 | -2.38 |
| AC2^ToLCPalV^ | 73 | 3 | 174 | 219 | 60/31/3 | 0.991 | 0.056 | 23.26 | 56 | 0.087 | -1.67 | -3.38 |
| AC3^ToLCPalV^ | 73 | 6 | 172 | 225 | 65/39/5 | 0.982 | 0.067 | 26.85 | 52 | 0.088 | -1.45 | -2.41 |
| AC4^ToLCPalV^ | 73 | 2 | 82 | 112 | 18/15/2 | 0.898` | 0.039 | 6.63 | 35 | 0.099 | -2.42 | -4.79 |
| AV1^ToLCPalV^ | 73 | 1 | 326 | 439 | 153/64/18 | 0.995 | 0.093 | 71.62 | 64 | 0.088 | -0.72 | -1.38 |
| AV2^ToLCPalV^ | 73 | 1 | 113 | 146 | 58/25/4 | 0.985 | 0.093 | 29.03 | 51 | 0.067 | -0.11 | -0.27 |
| BV1^ToLCPalV^ | 46 | 15 | 339 | 434 | 199/66/14 | 0.986 | 0.095 | 76.22 | 34 | 0.096 | -0.83 | 0.17 |
| BC1^ToLCPalV^ | 46 | 16 | 289 | 383` | 173/57/18 | 0.982 | 0.086 | 73.11 | 33 | 0.077 | -0.59 | 0.52 |
| AC1^WmCSV^ | 37 | 3 | 126 | 148 | 53/13/2 | 0.986 | 0.019 | 21.11 | 28 | 0.028 | -1.57 | -1.74 |
| AC2^WmCSV^ | 37 | 0 | 53 | 63 | 17/6/1 | 0.956 | 0.023 | 9.70 | 24 | 0.031 | -1.30 | -2.54 |
| AC3^WmCSV^ | 37 | 0 | 48 | 57 | 12/8/0 | 0.929 | 0.019 | 7.93 | 22 | 0.029 | -1.52 | -2.71 |
| AC4^WmCSV^ | 37 | 0 | 8 | 9 | 3/0/0 | 0.468 | 0.004 | 0.67 | 8 | 0.013 | -1.87 | -2.02 |
| AC5^WmCSV^ | 37 | 0 | 102 | 110 | 36/3/1 | 0.974 | 0.015 | 12.04 | 27 | 0.032 | -2.01 | -3.02 |
| AV1^WmCSV^ | 37 | 3 | 117 | 128 | 40/6/1 | 0.977 | 0.018 | 14.57 | 27 | 0.036 | -1.95 | -2.97 |
| AV2^WmCSV^ | 37 | 0 | 46 | 50 | 18/2/0 | 0.965 | 0.013 | 4.91 | 24 | 0.031 | -2.16 | -2.55 |
| BV1^WmCSV^ | 31 | 0 | 150 | 168 | 63/14/1 | 0.976 | 0.034 | 25.75 | 26 | 0.050 | -1.48 | -1.65 |
| BC1^WmCSV^ | 31 | 0 | 173 | 199 | 85/19/2 | 0.984 | 0.036 | 33.09 | 24 | 0.048 | -1.32 | -1.20 |

Abbreviations: Number seq - No of sequences used for the analysis; InDel sites - Insertion and deletions; S - Number of polymorphic (segregating) site; Eta (h) - Total number of mutations; Number of variants - Variable (polymorphic) sites (two/three/four variants); h - Number of Haplotypes; Hd - Haplotype (gene) diversity; Pi - Nucleotide diversity (per site); k - Average number of nucleotide differences

**Supplementary Table 5:** Estimation of selection pressure on the ORFs encoded by TYLCV, ToLCPalV (DNA-A and DNA-B), and WmCSV (DNA-A and DNA-B)

| **ORF** | **Best-fit model** | **Mean distance (d)** | **dN** | **dS** | **dN/dS** | **FUBAR Posterior probability (p≤0.9)** | | **SLAC (p≤0.05)** | |
| --- | --- | --- | --- | --- | --- | --- | --- | --- | --- |
|  |  |  |  |  |  | **PS** | **NS** | **PS** | **NS** |
| C1^TYLCV^ | K2+G | 0.088±0.010 | 0.063±0.010 | 0.349±0.050 | 0.180 | 0 | 130 | 0 | 59 |
| C2^TYLCV^ | JC+G | 0.105±0.019 | 0.066±0.015 | 0.177±0.051 | 0.373 | 5 | 13 | 0 | 6 |
| C3^TYLCV^ | JC+G | 0.076±0.018 | 0.057±0.013 | 0.136±0.043 | 0.419 | 5 | 20 | 0 | 11 |
| C4^TYLCV^ | JC | 0.061±0.006 | 0.213±0.031 | 0.124±0.039 | 1.718 | 12 | 3 | 0 | 0 |
| V1^TYLCV^ | JC | 0.011±0.003 | 0.012±0.004 | 0.128±0.028 | 0.094 | 1 | 47 | 0 | 21 |
| V2^TYLCV^ | K2 | 0.043±0.004 | 0.018±0.007 | 0.118±0.038 | 0.152 | 3 | 8 | 0 | 1 |
| AC1^ToLCPalV^ | K2+G | 0.054±0.010 | 0.038±0.007 | 0.277±0.040 | 0.137 | 4 | 156 | 2 | 104 |
| AC2^ToLCPalV^ | K2+G | 0.083±0.017 | 0.053±0.012 | 0.081±0.030 | 0.654 | 6 | 12 | 0 | 9 |
| AC3^ToLCPalV^ | JC+G | 0.063±0.010 | 0.048±0.013 | 0.182±0.046 | 0.264 | 0 | 31 | 0 | 23 |
| AC4^ToLCPalV^ | JC | 0.17±0.004 | 0.049±0.027 | 0.041±0.016 | 1.195 | 4 | 3 | 0 | 1 |
| AV1^ToLCPalV^ | JC+G | 0.041±0.008 | 0.427±0.027 | 20.3±4.25 | 0.021 | 1 | 123 | 0 | 90 |
| AV2^ToLCPalV^ | JC+G | 0.104±0.020 | 0.062±0.015 | 0.214±0.056 | 0.29 | 4 | 20 | 0 | 15 |
| BV1^ToLCPalV^ | T92+G+I | 0.108±0.007 | 0.108±0.014 | 0.101±0.023 | 1.07 | 1 | 120 | 0 | 55 |
| BC1^ToLCPalV^ | HKY+G | 0.095±0.005 | 0.092±0.012 | 0.077±0.020 | 1.19 | 1 | 124 | 0 | 64 |
| AC1^WmCSV^ | JC+G | 0.013±0.003 | 0.009±0.003 | 0.057±0.015 | 0.158 | 2 | 27 | 0 | 1 |
| AC2^WmCSV^ | JC+G | 0.021±0.008 | 0.017±0.006 | 0.046±0.021 | 0.369 | 3 | 6 | 0 | 2 |
| AC3^WmCSV^ | JC | 0.017±0.006 | 0.017±0.007 | 0.030±0.017 | 0.567 | 4 | 3 | 0 | 0 |
| AC4^WmCSV^ | JC | 0.003±0.003 | 0.005±0.004 | 0.003±0.002 | 1.667 | 0 | 0 | 0 | 0 |
| AC5^WmCSV^ | JC | 0.031±0.006 | 0.017±0.005 | 0.009±0.005 | 1.889 | 4 | 1 | 0 | 0 |
| AV1^WmCSV^ | JC+G | 0.012±0.003 | 0.009±0.003 | 0.054±0.017 | 0.167 | 3 | 23 | 0 | 11 |
| AV2^WmCSV^ | JC | 0.018±0.004 | 0.013±0.006 | 0.017±0.012 | 0.765 | 3 | 4 | 0 | 1 |
| BV1^WmCSV^ | K2+G | 0.035±0.004 | 0.018±0.005 | 0.090±0.002 | 0.2 | 1 | 127 | 0 | 2 |
| BC1^WmCSV^ | K2+G | 0.038±0.003 | 0.015±0.004 | 0.110±0.023 | 0.136 | 1 | 124 | 0 | 3 |

The table employs the following abbreviations: FUBAR (Fast, Unconstrained Bayesian Approximation), SLAC (single-likelihood ancestor counting), dN (non-synonymous), dS (synonymous), T92 (Tamura 3-parameter), K2 (Kimura 2-parameter), G (Gamma distribution), PS (positively selected sites), and NS (negatively selected sites).

**Supplementary Table 6:** Mean substitution and codon position mutation rate of TYLCV, ToLCPalV (DNA-A and DNA-B), and WmCSV (DNA-A and DNA-B)

| **Dataset** | **Clock type** | **Mean nt substitution rate (site^-1^ year^-1^** | **At 95% HPD interval** |
| --- | --- | --- | --- |
| TYLCV | Strict | 1.02E-2 (6) | 6.28E-2, 14.82E-2 |
|  | Relaxed | 4.27E-2 (25) | 3.05E-2, 5.33E-2 |
| ToLCPalV DNA-A | Strict | 4.51E-2 (7) | 4.05E-3, 24.8E-2 |
|  | Relaxed | 8.8E-2 (4) | 4.78E-2, 12.21E-2 |
| ToLCPalV DNA-B | Strict | 4.26E-2 (9) | 8.86E-2, 12.01E-2 |
|  | Relaxed | 4.55E-2 (17) | 2.11E-2, 6.75E-2 |
| WmCSV_DNA-A | Strict | 3.06E-2 (13) | 1.87E-2, 4.38E-2 |
|  | Relaxed | 9.48E-3 (22) | 5.43E-3, 2.05E-2 |
| WmCSV_DNA-B | Strict | 8.2E-2 (9) | 4.84E-2, 12.89E-2 |
|  | Relaxed | 2.73E-2 (30) | 1.53E-2, 4.16E-2 |

The values of effective sample size (ESS) are provided within parentheses.

**Supplementary Table 7:** Codon position mutation rate of the ORFs encoded by TYLCV, ToLCPalV (DNA-A and DNA-B), and WmCSV (DNA-A and DNA-B)

| **ORFs** | **Clock type** | **Mean nt substitution rate (site^-1^ year^-1^** | **At 95% HPD interval** | **CoP1 mut** | **CoP2 mut** | **CoP3 mut** |
| --- | --- | --- | --- | --- | --- | --- |
| C1^TYLCV^ | Strict | 2.06E-2 (3) | 8.7E-2, 42.39E-2 | 0.575 (70) | 0.581 (64) | 1.54 (74) |
|  | Relaxed | 6.49E-2 (5) | 4.14E-2, 8.84E-2 | 0.375 (70) | 1.565 (76) | 0.76 (32) |
| C2^TYLCV^ | Strict | 12.4E-2 (4) | 4.26E-2 26.52E-2 | 0.567 (49) | 1.845 (58) | 0.608 (89) |
|  | Relaxed | 3.01E-2 (12) | 1.67E-2, 5.26E-2 | 0.487 (76) | 0.336 (91) | 2.17 (91) |
| C3^TYLCV^ | Strict | 15.5E-2 (4) | 7.53E-2, 31.42E-2 | 0.494 (32) | 0.366 (17) | 2.14 (17) |
|  | Relaxed | 3.35E-2 (16) | 2.09E-2, 4.48E-2 | 0.844 (76) | 0.592 (73) | 1.86 (94) |
| C4^TYLCV^ | Strict | 18.11E-2 (3) | 7.15E-2, 37.7E-2 | 0.729 (21) | 0.376 (5) | 1.69 (5) |
|  | Relaxed | 5.16 (8) | 3.17E-2, 13.01E-2 | 0.876 (74) | 0.3154 (91) | 1.39 (47) |
| V1^TYLCV^ | Strict | 9.71E-2 (4) | 3.92E-2, 18.6E-2 | 0.574 (32) | 0.267 (17) | 1.45 (27) |
|  | Relaxed | 2.19E-2 (24) | 1.44E-2, 2.97E-2 | 1.076 (44) | 0.56 (91) | 1.47 (47) |
| V2^TYLCV^ | Strict | 5.78E-2 (5) | 2.22E-2, 12.3E-2 | 0.574 (32) | 0.467 (17) | 1.24 (17) |
|  | Relaxed | 1.47E-2 (7) | 7.72E-3, 3.26E-2 | 1.09 (44) | 0.6154 (91) | 1.28 (47) |
| AC1^ToLCPalV^ | Strict | 9.44E-3 (5) | 5.0469E-4, 2.07E-2 | 0.6471 (55) | 0.436 (57) | 1.917 (67) |
|  | Relaxed | 7.11E-2 (4) | 2.68E-2, 1.93E-2 | 0.661 (30) | 0.429 (91) | 1.91 (52) |
| AC2^ToLCPalV^ | Strict | 18.98E-2 (11) | 1.41E-2, 78.1E-2 | 1.163 (91) | 0.74 (63) | 1.09 (91) |
|  | Relaxed | 2.21E-2 (3) | 5.16E-3, 5.31E-2 | 1.26 (10) | 0.718 (91) | 1.023 (16) |
| AC3^ToLCPalV^ | Strict | 87.17E-2 (6) | 7.6E-2, 2.07 | 0.775 (91) | 0.681 (64) | 1.54 (74) |
|  | Relaxed | 4.46E-2 (8) | 1.6E-2, 9.48E-2 | 0.716 (63) | 0.7 (91) | 1.58 (66) |
| AC4^ToLCPalV^ | Strict | 29.8E-2 (4) | 2.6E-2, 56.5E-2 | 0.475 (60) | 1.765 (67) | 0.76 (22) |
|  | Relaxed | 77.7E-2 (7) | 17.52E-2, 2.29 | 0.467 (49) | 1.825 (58) | 0.708 (89) |
| AV1^ToLCPalV^ | Strict | 33.11 (6) | 33.46E-2, 118.07 | 0.487 (76) | 0.336 (91) | 2.17 (91) |
|  | Relaxed | 13.3E-2 (14) | 7.68E-2, 19.79E-2 | 0.494 (32) | 0.366 (17) | 2.14 (17) |
| AV2^ToLCPalV^ | Strict | 8.21E-2 (3) | 5.64E-4, 27.51E-2 | 0.944 (76) | 0.392 (73) | 1.66 (94) |
|  | Relaxed | 11.9E-2 (4) | 2.43E-2, 24.4E-2 | 0.929 (21) | 0.476 (5) | 1.59 (5) |
| BV1^ToLCPalV^ | Strict | 9.09E-2 (6) | 5.22E-4, 15.19E-2 | 0.744 (56) | 0.292 (73) | 1.66 (94) |
|  | Relaxed | 6.46E-2 (6) | 2.6E-2, 8.48E-2 | 0.516 (63) | 0.8 (91) | 1.48 (66) |
| BC1^ToLCPalV^ | Strict | 7.50E-2 (6) | 3.71E-2, 11.51E-2 | 0.824 (55) | 0.492 (45) | 1.46 (74) |
|  | Relaxed | 3.46E-2 (6) | 2.8E-2, 7.48E-2 | 0.416 (52) | 0.6 (91) | 2.58 (66) |
| AC1^WmCSV^ | Strict | 1.74E-2 (4) | 7.3895E-3, 3.21E-2 | 0.676 (74) | 0.5154 (91) | 1.3976 (47) |
|  | Relaxed | 4.64E-2 (7) | 2.53E-2, 7.01E-2 | 0.474 (32) | 0.367 (17) | 1.14 (17) |
| AC2^WmCSV^ | Strict | 1.55E-2 (3) | 6.59E-3, 2.68E-2 | 1.0869 (44) | 0.5154 (91) | 1.3976 (47) |
|  | Relaxed | 4.12E-2 (4) | 1.87E-2, 6.8E-2 | 0.816 (53) | 0.653 (87) | 1.48 (63) |
| AC3^WmCSV^ | Strict | 1.51E-2 (4) | 5.60E-3, 3.83E-2 | 0.754 (63) | 0.416 (91) | 1.784 (47) |
|  | Relaxed | 3.76E-2 (5) | 1.56E-2, 7.22E-2 | 0.675 (91) | 0.581 (54) | 1.34 (64) |
| C4^WmCSV^ | Strict | 4.82E-03 (7) | 9.42E-4, 1.14E-2 | 0.325 (73) | 0.523 (82) | 1.278 (56) |
|  | Relaxed | 1.14E-2 (5) | 3.12E-3, 2.15E-2 | 0.467 (59) | 1.825 (68) | 0.708 (89) |
| AC5^WmCSV^ | Strict | 2.25E-2 (5) | 9.4E-3, 4.1E-2 | 1.754 (53) | 0.216 (91) | 0.547 (47) |
|  | Relaxed | 5.35E-2 (8) | 3.02E-2, 8.63E-2 | 1.57 (91) | 0.74 (63) | 1.43 (91) |
| AV1^WmCSV^ | Strict | 6.56E-2 (11) | 1.18E-3, 21.38E-2 | 0.694 (81) | 0.487 (65) | 1.89 (76) |
|  | Relaxed | 4.79E-2 (4) | 2.35E-2, 73.33E-2 | 0.691 (75) | 0.5 (91) | 1.80 (49) |
| AV2^WmCSV^ | Strict | 2.28E-2 (3) | 7.11E-3, 4.24E-2 | 0.368 (63) | 0.876 (51) | 0.489 (47) |
|  | Relaxed | 5.19E-2 (4) | 2.04E-2 8.39E-2 | 0.425 (73) | 0.623 (82) | 1.278 (56) |
| BV^WmCSV^ | Strict | 4.88E-2 (6) | 2.48E-2, 8.22E-2 | 0.466 (61) | 0.875 (53) | 0.489 (57) |
|  | Relaxed | 2.35E-2 (6) | 4.02E-2, 9.63E-2 | 2.57 (91) | 0.84 (62) | 1.44 (93) |
| BC1^WmCSV^ | Strict | 5.54E-2 (6) | 2.98E-2, 8.92E-2 | 0.368 (43) | 0.876 (62) | 0.489 (93) |
|  | Relaxed | 2.65E-2 (8) | 2.02E-2, 9.63E-2 | 2.47 (91) | 0.74 (74) | 1.52 (84) |

The table employs abbreviations such as codon position mutation (CoP mut) and highest probability density (HPD). The effective sample size (ESS) values are provided in parentheses
